# The Temple Grandin Genome: Comprehensive Analysis in a Scientist with High-Functioning Autism

**DOI:** 10.3390/jpm11010021

**Published:** 2020-12-29

**Authors:** Rena J. Vanzo, Aparna Prasad, Lauren Staunch, Charles H. Hensel, Moises A. Serrano, E. Robert Wassman, Alexander Kaplun, Temple Grandin, Richard G. Boles

**Affiliations:** 1 Lineagen, Inc., Salt Lake City, UT 84109, USA; aparna.ambastha@gmail.com (A.P.); laurenstaunch@gmail.com (L.S.); ghensel3@comcast.net (C.H.H.); mserrano@lineagen.com (M.A.S.); drwassman@icloud.com (E.R.W.); 2 Variantyx, Inc., Framingham, MA 01701, USA; alex.kaplun@variantyx.com; 3 Department of Animal Science, Colorado State University, Fort Collins, CO 80523, USA; Cheryl.Miller@ColoState.EDU; 4 The Center for Neurological and Neurodevelopmental Health, Voorhees, NJ 08043, USA; drboles@molecularmito.com

**Keywords:** autism spectrum disorder, genetic testing, chromosomal microarray analysis, whole exome sequencing, whole genome sequencing, clinical utility, polygenic risk scores, Temple Grandin

## Abstract

Autism spectrum disorder (ASD) is a heterogeneous condition with a complex genetic etiology. The objective of this study is to identify the complex genetic factors that underlie the ASD phenotype and other clinical features of Professor Temple Grandin, an animal scientist and woman with high-functioning ASD. Identifying the underlying genetic cause for ASD can impact medical management, personalize services and treatment, and uncover other medical risks that are associated with the genetic diagnosis. Prof. Grandin underwent chromosomal microarray analysis, whole exome sequencing, and whole genome sequencing, as well as a comprehensive clinical and family history intake. The raw data were analyzed in order to identify possible genotype-phenotype correlations. Genetic testing identified variants in three genes (*SHANK2*, *ALX1*, and *RELN*) that are candidate risk factors for ASD. We identified variants in *MEFV* and *WNT10A*, reported to be disease-associated in previous studies, which are likely to contribute to some of her additional clinical features. Moreover, candidate variants in genes encoding metabolic enzymes and transporters were identified, some of which suggest potential therapies. This case report describes the genomic findings in Prof. Grandin and it serves as an example to discuss state-of-the-art clinical diagnostics for individuals with ASD, as well as the medical, logistical, and economic hurdles that are involved in clinical genetic testing for an individual on the autism spectrum.

## 1. Introduction

Autism spectrum disorder (ASD) is one of the most common neurodevelopmental disorders characterized by impairments in communication and social interaction and the presence of restrictive and repetitive behaviors [1]. The American College of Medical Genetics and Genomics (ACMG) recommends genetic evaluation for individuals with ASD [2]. Discovering the underlying genetic cause for ASD can improve the care and management by personalizing services and treatment, including addressing the medical risks that are associated with the genetic diagnosis [3]. We performed chromosomal microarray (CMA), as well as whole exome and genome sequencing (WES, WGS), on our co-author, Prof. Temple Grandin (T.G.), a widely recognized animal scientist and woman with high-functioning ASD, who is renowned for her insights on the condition. While using this report of the genomic findings in T.G., we create a discourse on state-of-the-art diagnostics for individuals with ASD. Predictably, we found many variants of uncertain significance (VUSs). However, we also identified the variants that were previously reported in the literature as pathogenic/disease-causing and overlap with her clinical features. Furthermore, many of these variants lie in genes that will personalize medical management and guide potential therapeutic options, which underscores the importance of clinical genetic testing in those with ASD. 

## 2. Materials and Methods

### 2.1. Case Report

T.G. was a full-term female infant born in 1947 after an uncomplicated pregnancy, labor, and delivery. She had normal muscle tone and early motor milestones. However, she did not make eye contact and had touch sensitivity and aversion, including stiffening up when held by others. Even based on these specific features, ASD was not suggested as the diagnostic entity had only been described four years previously [4]. A neurologist performed an electroencephalogram and ruled out petite mal epilepsy, and a hearing test was normal. T.G. was diagnosed with “minimal brain damage” at two years old. At two and a half years old, expressive and receptive language delays were noted (she was non-verbal), and she was enrolled in speech therapy. With intensive treatment and emphasis on turn-taking games, she began speaking at age three and a half and was fully verbal by age four. She maintained typical autistic behaviors, such as repetitively dribbling sand through her hands at the beach and tantrums after sudden loud noises. The associated issues included stuttering and challenges with auditory and sensory integration. In particular, touch aversion improved with the use of a squeezing machine during childhood, which T.G. designed and has adapted in order to improve ethical animal husbandry management [5]. She also reported difficulties with social interactions in childhood and adolescence. The diagnosis of autism came later in elementary school by a psychiatrist. At age nine years and at age twelve years, her IQ was tested with the Wechsler. Her full-scale IQ was 120 on the first test and 137 on the second test. The measurement of cognitive abilities ranged from disability to gifted; for example, cognitive skills that require visualization, including block design and puzzle completion, were superior, whereas auditory integration was reduced [5,6]. T.G. received a Ph.D. and has been highly successful in her career regarding animal behavior-informed agriculture design prior to her career lecturing on ASD. Despite the absence of formal testing for ASD (based on age), T.G. meets current diagnostic criteria for ASD, given her childhood clinical history. The family history is unremarkable for specific diagnoses of ASD or intellectual disability in other family members. However, T.G.’s maternal grandmother was medicated for anxiety, and T.G. suspects that her father had high-functioning ASD. Both of T.G.’s parents held college degrees, and her maternal grandfather had particular academic success as an MIT-trained engineer and co-inventor of the autopilot for airplanes. The remainder of the family history is non-contributory without a suggested inheritance pattern; further details on family history is withheld in order to maintain privacy. 

T.G. reports a substantial, decades-long history of chronic myalgia, muscle rigidity, paresthesia, and hyperesthesia of the feet, as well as sudden episodes of feeling “boiling hot”. She reports coordination difficulties and tires easily with exercise, reportedly since childhood. She experiences insomnia and requires physical exercise at bedtime (“100 sit-ups”) to sleep. Severe anxiety and panic attacks have been major life-long issues that have moderately improved on desipramine (50 mg/day since 1980). Desipramine also alleviated colitis-like symptoms. Her diet is high in animal protein, and its reduction or elimination results in perceived irritability. A peculiar rash with eczema- and psoriasis-like features, which had been present since early childhood and diagnosed in adolescence as eczema, responds to topical steroids. She has a widow’s peak (a V-shaped growth of hair in the center of the forehead). Cranial MRI identified the asymmetry of the ventricles and a reduction in cerebellum size [7]. 

T.G. has microdontia and hypodontia, including six missing adult teeth (two on bottom, four on top, bilaterally symmetrical) which are absent on x-ray; she did not lose the corresponding deciduous teeth until the third to sixth decades. Two dentists have commented that she has a high arched palate. Additional ectodermal dysplasia (ED)-related manifestations include soft and very brittle nails, hyperhidrosis, and body hair loss since the fifth decade of life. 

### 2.2. Sample and Genetic Analysis

We obtained written consent from T.G. to disclose her name and health information for this study and publication. We did not have access to parental samples. Genomic DNA was extracted from an oral swab (OC-100Dx, DNA Genotek, Kantana, ON, Canada) using the PureGene extraction kit (Qiagen, Inc., Valencia, CA, USA). DNA extraction and all the analyses were performed in CAP and CLIA certified laboratories. Table 1 presents details regarding the various genetic testing technologies utilized in this study. 

## 3. Results

In our testing population on the aforementioned custom array, we have historically observed that 28% of patients with neurodevelopmental disorders have one or more abnormal or potentially abnormal copy number variants (CNVs) [11]. In the case of T.G., we did not identify any pathogenic or likely pathogenic CNVs on either custom CMA or WGS, as reported through our clinical pipeline and ACMG reporting criteria [12], and the results were consistent with a normal female chromosome complement. However, out of over 4000 structural variants of different types (including deletions, duplications, inversions, LOH, break points, and insertions of transposable elements; see Table S2 for complete list) some of the variants of unknown significance could be relevant to patient’s phenotype given their relevance to brain pathology. Two of these variants are discussed in more detail below, both being located on the q arm of chromosome 9.

One of them, a heterozygous duplication of chr9q34.3q34.3x3(138,014,000–138,228,000) is about 200 kbp long and it includes several noncoding genes and exons 19 to 47 of a calcium channel gene CACNA1B. *CACNA1B* is associated with Neurodevelopmental disorder with seizures and nonepileptic hyperkinetic movements, according to OMIM. The disease is autosomal recessive, and while the deletion has a 0.00035 allele frequency in general population (four cases out of 11,295 in DGV database), one cannot completely exclude mild phonotype in heterozygotes.

Another candidate structural variant is a 1656 bp heterozygous deletion of chr9q34.13q34.13x1(131,153,102–131,154,758), which is not found in the general population. The deletion affects exon 3 of non-coding gene RP11-544A12.4. Interestingly, this gene overlaps *NUP214*, which is located on the opposite DNA strand and, according to OMIM, is associated with susceptibility to acute infection-induced encephalopathy-9. However, the facts that disease is recessive and for *NUP214* the deletion is entirely intronic suggest that this variant is a less feasible candidate to be causative, at least not by itself.

WGS interpretation also revealed that CGG repeats that correspond to fragile X syndrome are 30,30 (a frequent normal genotype). 

Three sequence variants of interest were identified in suspected or known ASD risk genes SHANK2, ALX1, and RELN (Table 2). Nevertheless, as of November 2020, none of these variants met the ACMG guidelines for “pathogenic” or “likely pathogenic” designation and, thus, are clinically classified as variants of uncertain significance (VUS) [12]. A heterozygous missense variant in *SHANK2* (p.H64R) was identified. This missense variant is a change from histidine to arginine. The histidine at this location does not lie in any well-defined protein domains. However, histidine is present at this location in primates. Further, the substitution of histidine to arginine is predicted by SIFT to be deleterious. This suggests that the his to arg amino acid substitution may alter protein function. Additionally, the variant is only observed in seven of 184,874 reference alleles (allele frequency: 3.79 × 10^−5^) in the Genome Aggregation Database, a database of approximately 141,000 individuals without severe genetic conditions (gnomAD) [13,14]. Several studies suggest a role for *SHANK2* in ASD and/or intellectual disability (ID). In one publication, a patient with ASD harbored a *de novo* nonsense variant in *SHANK2*, while two additional, unrelated patients with ASD and mild-to-moderate intellectual disability had *de novo* deletions in *SHANK2* [15]. This study suggests that the haploinsufficiency of the SHANK2 gene may affect synaptic function and predispose to ASD and/or ID. In a subsequent study, a novel *de novo SHANK2* deletion was identified in another patient with ASD. Further, sequencing identified a significant enrichment of variants affecting conserved amino acids in *SHANK2* (3.4% of autism cases and 1.5% of controls, *P* = 0.004, OR = 2.37) [16]. In neuronal cell cultures, the variants that were identified in patients were associated with reduced synaptic density at dendrites when compared to variants that were only detected in controls. Interestingly, the three patients with *de novo* deletions identified in the two aforementioned studies also carry inherited CNVs at 15q11-q13 previously associated with neuropsychiatric disorders [17,18,19]. These data strengthen the role of synaptic gene dysfunction in ASD and support the "multiple hit model", suggesting that a better knowledge of these genetic interactions will be important in understanding the complex inheritance pattern of ASD [18,20]. 

A heterozygous missense variant (p.R64L) was identified in the ALX1 gene. *ALX1* encodes a transcription factor that plays a role in development, including proper neural crest migration in animal models [21]. Bi-allelic loss-of-function variants in *ALX1* cause frontonasal dysplasia, while gain-of-function variants are hypothesized to impact neurodevelopment [22,23]. T.G.’s craniofacial finding of a “widow’s peak” could be related to this variant, as neural crest cells can be found in hair follicles [24]. This variant was found in 1203 of the 280,730 reference alleles (allele frequency: 0.004285) in the gnomAD database [14]. Additionally, the variant identified in T.G. was one of several potential ASD risk variants that were identified in two unrelated multiplex families [25]. In one family, this variant was shared by two siblings with ASD and it was inherited from their unaffected father. In the second family, the variant was found in an individual with ASD and it was not found in his two brothers with ASD, but was inherited from his unaffected father. Importantly, reportedly unaffected parents were not phenotyped in detail in that publication. The authors showed the *ALX1* variant was observed multiple times in their population study (27/1541 cases and 58/5785 controls), yielding an odds ratio of 1.75 (95% confidence interval 1.11 to 2.77; *p* = 0.022; on page 7 of Matsunami et al., 2014 [25]). Although this specific variant has been observed in a supposedly unaffected control population, its higher prevalence in individuals with ASD when compared to those without ASD supports it as a potential risk factor. Further research is needed in order to confirm the impact of this variant on gene function and the role of *ALX1* in ASD susceptibility. 

A heterozygous missense variant in the RELN gene (p.T1002S) was also identified in T.G. *RELN* encodes the reelin protein, which is thought to control interactions between cells for cell positioning and neuronal migration. Although the serine for threonine substitution is conservative and does not lie within any known protein domain, this variant was not present in the gnomAD database and it is conserved across different vertebrate species, except lamprey (PhyloP 1.048) [14]. Variants in *RELN* are associated with autosomal recessive lissencephaly with cerebellar hypoplasia (OMIM). In addition, *de novo* variants in *RELN* have been observed in individuals with ASD in several studies [26,27,28]. 

*MEFV* is a fourth gene that harbored variants with clinical overlap for T.G. Two heterozygous variants, p.R408Q and p.P369S, were identified in T.G. and they have been reported to be disease-associated. Allele frequencies in the gnomAD database are 0.00001595 (four out of 250,794 reference alleles) and 0.01470 (4150 out of 282,228 reference alleles, respectively [14]. The MEFV gene encodes a protein, called pyrin, whose function is not fully understood, but appears to direct the migration of white blood cells to sites of inflammation and downregulate the inflammatory response following the improvement of infection or injury. Over 80 variants in *MEFV* have been associated with familial Mediterranean fever (FMF), a highly complex and variable condition that can exhibit either autosomal dominant or autosomal recessive inheritance [29]. Studies suggest these variants are in linkage disequilibrium and are, thus, in cis [30]; indeed, a review of the WGS read data that were utilized in this study (2 × 150 bp) was long enough to confirm cis phasing.]. Despite having ClinVar associations that range from “benign” to “pathogenic”, these variants, when found together, have been published as associated with disease and they are often included in clinical gene panels that are designed to test for FMF [31,32]. Most of the patients with both variants are reported to have an atypical clinical presentation. Although T.G. does not strictly meet the Tel-Hashomer clinical criteria for FMF, she has symptoms that are consistent with the atypical presentation of the condition seen in those with the same genotype, including frequent intermittent hot spells, muscles that are stiff and sore, episodes of calor, and paresthesia in both feet, and lifelong skin rashes that are diagnosed as eczema [32]. FMF has not been reported to be associated with ASD; however, inflammation is one of many pathways implemented in ASD pathogenesis and, thus, we cannot exclude these variants as being risk factors for ASD in T.G. 

Further, *WNT10A* is another gene harboring a variant with overlap to T.G.’s phenotype. A homozygous variant (p.F228I) has been previously reported as pathogenic. *WNT10A* is a member of the WNT gene family, which encodes proteins that are implicated in several developmental processes, including the regulation of cell fate and patterning during embryogenesis [33]. Although p.F228I is a conservative amino acid substitution, the amino acid at this position is conserved across different species (PhyloP 0.964) and the variant has been predicted to be deleterious to the protein structure or function by in silico prediction tools. The variant identified here has been reported previously, either in the homozygous state or in trans with a second pathogenic variant, in individuals with either isolated oligodontia, tooth agenesis, or with other features of ED [34,35,36,37,38]. The p.F228I variant that is identified in T.G. is observed at a relatively high frequency in the general population (heterozygous carrier frequency: 0.0137, homozygote frequency: 0.000153 in the gnomAD database) [14]. Oligodontia is observed in approximately 0.14% of the population and, in one study, it was shown that variants in *WNT10A* were present in more than half of the cases of isolated oligodontia [38,39]. The variant in WNT10A possibly explains the multiple ED-like manifestations in T.G. involving her teeth, nails, hair, and sweat glands.

Please see Supplementary Table S1 for additional variants, which were identified in both the whole exome and whole genome sequencing assays that were run independently, with additional clinical overlap that potentially contributes to T.G.’s ASD, sleep pathogenesis, anxiety, mitochondrial function, and more, including some with potential therapeutic targets. 

From a proactive standpoint, sequencing also identified a *CYP2C9* genotype that was associated with slowed metabolism of many drugs, including the anticoagulant warfarin [40]. This information, coupled with T.G.’s heterozygous status of the *VKORC1* 1639G>A variant also revealed by this testing, provides specific dosing information if warfarin is prescribed to reduce the risk adverse events. Furthermore, this *CYP2C9* genotype is also correlated with slowed metabolism of the anti-epileptic drug phenytoin, which would impact the dosing recommendations; this is critical information, given that there is a high rate of comorbidity between epilepsy/seizures and ASD [41,42].

Given the presence of multiple genetic variants potentially contributing to the manifestation of ASD in T.G., we sought to explore her data through a currently available polygenic risk score (PRS) algorithm (impute.me). This algorithm showed that T.G.’s PRS for ASD is lower than 99% and greater than 1% of the general population by assessing 17 single nucleotide polymorphisms (SNPs) that were previously reported to be associated with ASD [43].

## 4. Discussion

We present this case of a female scientist, Prof. Temple Grandin, with high-functioning ASD and other clinical sequelae, who was referred for clinical diagnostic testing. Through various test methodologies, we identified variants of unknown significance in three ASD risk genes (*SHANK2*, *ALX1*, and *RELN*) and other variants that impact genes that are possibly relevant to ASD pathology. This supports the concept of a polygenic model in ASD. Surprisingly, the PRS model used for T.G. showed her risk for ASD in the 1st centile of the general population. The tool did provide a pie chart indicating that the genetic liability captured by this assessment is very small (~1%), which echoes the disclaimer supplied in other publications regarding the limitations of PRS for clinical application [44]. There are several potential reasons for the contradictory ASD PRS score in T.G., given her clinical diagnosis. The SNPs that were used to generate the score in the impute.me tool were derived from case cohort individuals diagnosed with ASD prior to 2014 [43]. Importantly, Asperger disorder was a diagnostic entity until 2013, according to the American Psychiatric Association’s Diagnostic and Statistical Manual of Mental Disorders (DSM). Therefore, the case cohort whose data are represented in the tool is likely more representative of those individuals who are lower functioning, which is in stark contrast to the phenotype of T.G. Additionally, we, as a community, do not yet know all of the genes and variants contributing to ASD. Additionally, finally, some co-occurring traits in those with ASD are also clearly polygenic (IQ and anxiety for instance) and may skew the algorithmic outcomes. In summary, a low PRS score, at least for ASD, cannot be used to rule out the potential for a future clinical diagnosis.

While T.G. herself does not harbor any known, identifiable ASD-related variants that garner specific medical management changes, this is a possibility for others with ASD and genetic testing should be pursued as a standard of care in line with ACMG and other medical guidelines [45]. Importantly, genetic testing did identify actionable variants that contribute to T.G.’s clinical symptomatology and can be specifically addressed in order to improve her functional symptoms and prevent further medical issues. Of note, prior to testing, additional neurological and non-neurological symptoms were attributed to a broader diagnosis of ASD and not specifically addressed based on underlying genetic etiology. This is critical for medical action, as well as family understanding, coping, and improved quality of life. 

For example, clinical features that disturb T.G.’s activities of daily living are longstanding calor and paresthesia in both feet. These correlate with the two identified *MEFV* variants that were reported in cases with atypical familial Mediterranean fever. FMF is a treatable condition, and T.G. has since been referred to see a specialist. In addition, the homozygous pathogenic variant in *WNT10A* is likely to explain T.G.’s multiple ED-related symptoms. Dentists and orthodontists incorporate specific management decisions for individuals with ED-related disorders. Fortunately, T.G. herself chose not to have dental implants; the avoidance of dental implants would have been advised previously had a diagnosis of ED been known at the time. Moreover, genetic testing has provided actionable guidelines for the future prescription of certain pharmacologic treatments that were impacted by *CYP2C9* metabolism. There are a variety of other findings for T.G. with currently less well-supported medical literature that will advance with time. For example, will some combination of CNVs and SNVs that are currently classified as benign emerge as a risk susceptibility for ASD? In the future, will the reclassification of VUS to pathogenic or likely pathogenic variants trigger medical action that prevents medical morbidity or mortality? A reinterpretation of the raw data and integration with medical care can be pursued based on T.G.’s medical course and preferences in the future. 

The power of clinically available genetic testing for those with ASD with or without co-occurring morbidities can be substantial for neurologic and non-neurologic precision medicine, as shown in this study. However, there are associated challenges that we must address as a medical community to make this process more impactful. One is a lack of trained clinical experts that are comfortable in reading vast amounts of genetic data and translating it in order to inform disease-associated factors and treatment options. Efforts should be made to train physicians and other healthcare providers in the practical use of genomics, as the future of health care depends on its understanding and application, including the limitations of using PRS models to predict future presence and severity for ASD. A second challenge is that the feasibility of a “genomics board” (akin to that of a multidisciplinary “tumor board”, which is standard in oncologic care) is hindered by state medical licensing and telemedicine laws. Genetic counselors (GCs) are essential in this process and growing in number, but they typically have long wait lists or their own state licensure barriers that encumber integrated care. Peer-to-peer consultation between physicians and GCs is not precluded by such laws; however, including the family in the discussion constitutes the practice of medicine in most jurisdictions. Third, market forces have resulted in low-cost exome and genome testing. However, careful report generation and detailed discussion with the patient’s attending providers takes substantial professional time and it is not practical under the current cost structures. Paradoxically, this results in healthcare providers being unaware of or unable to utilize the resulting complex genetic reports in order to improve clinical care and leads insurance companies to deny coverage for lack of clinical application. We propose that payer policies should be devised to commensurately compensate parties that are involved in sequencing and variant interpretation, as well as physicians and GCs for effective use of the data and the treatment of the patients. 

To summarize, these data support the concept that the genetic etiology of high-functioning ASD in T.G. could result from a combination of multiple genetic factors interacting in order to yield the observed clinical features. We demonstrated that comprehensive clinical phenotype information and genomics-trained providers/laboratorians are critical in the interpretation of genomic variants that were identified through these high-throughput genomic technologies. The genomic analysis that was carried out in this study provides a basis for at least part of T.G.’s clinical features and delivers suggestions for effective management of some of the symptoms. While improvements can be made to the process and application of genetic testing for individuals with ASD, it is effective and critical, as it currently exists for optimal medical and improved personal and family quality of life. 

## Figures and Tables

**Table 1:** Genetic testing methodologies utilized for Prof. Grandin in this study.

Genetic Test	Technology	Interpretation
Chromosomal microarray (CMA)	Custom-designed ^1^ Affymetrix microarray [8].	Chromosome Analysis Suite v2.0.1 software (Thermo Fisher Scientific, Santa Clara, CA)
Whole exome sequencing (WES)	Enrichment: Ion AmpliSeq^TM^ Exome Kit (Thermo Fisher Scientific) Sequencing: Ion Proton sequencing system (Thermo Fisher Scientific) with 200 bp amplicon read technology. Genome Reference Consortium Human Build 37 (GRCh37, hg19) using the Ion Torrent Suite software v4.2 (Thermo Fisher Scientific)	Clinical Sequence Analyzer tool from WuXi NextCode ^2^ (https://www.wuxinextcode.com) Non-sense, missense and splice site variants were analyzed and were assessed for predicted deleterious effects using the Variant Effect Predictor (VEP) score [9].
Whole genome sequencing (WGS)	2 × 150 bp reads on Illumina next-generation sequencing systems (mean coverage of 30x in the target region, including coding exons and 10 bp of flanking intronic sequence of the known protein-coding Ref-Seq genes) ^3^ Alignment to human reference genome hg19 and GRCh38	Primary data analysis: Illumina DRAGEN Bio-IT Platform v2.03, interpreted on internal proprietary software from Variantyx, Framingham, MA, USA. Secondary and tertiary data analysis: Internal laboratory systems and Biodiscovery’s NxClinical v4.3 or Illumina DRAGEN Bio-IT Platform v2.03 for CNV and absence of heterozygosity Detection and annotation of structural variants: The variants were called and annotated using Variantyx Genomic Intelligence structural variant pipeline [10].

Caption. ^1^ Affymetrix CytoScan-HD microarray plus 88,435 custom probes added to improve detection of copy number variants (CNVs) associated with neurodevelopmental disorders [8]. ^2^ Default settings used ^3^ >97% coverage of 22,000 genes in the genome at >30x.

**Table 2:** Rare missense variants that are potentially relevant to T.G.’s phenotype, identified on both whole exome and whole genome sequencing.

	Gene	Chromosome [GRCh37]	ISCN	HGVS Protein Reference	SIFT Predicted Effect	Clinical Classification	dbSNP/dbVar ID	gnomAD Allele Frequency (v2.1.1; GRCh37)	Genotype in T.G.	Symptoms with Overlap
1	*ALX1*	12: 85674230	NM_006982.3:c.191G>T	p.Arg64Leu	Deleterious	VUS	rs115596276	0.004285	Het	ASD
2	*RELN*	7: 103251145	NM_005045.4:c.3005C>G	p.Thr1002Ser	Tolerable	VUS	rs1376812440	NA	Het	ASD
3	*SHANK2*	11:70858182	NM_012309.4:c.191T>C	p.His64Arg	Deleterious	VUS	rs200995537	0.0000379	Het	ASD
4	*MEFV*	16: 3299468	NM_000243.3:c.1223G>A	p.Arg408Gln	Tolerable	Pathogenic	rs11466024	0.00001595	Het	FMF
5	*MEFV*	16: 3299586	NM_000243.3:c.1105C>T	p.Pro369Ser	Deleterious	Pathogenic	rs11466023	0.01470	Het	FMF
6	*WNT10A*	2:219755011	NM_025216.3:c.682T>A	p.Phe228Ile	Deleterious	Pathogenic	rs121908120	0.0137	Homo	ED

Caption. VUS-variant of uncertain clinical significance; NA-not available; Het-Heterozygous; Homo- Homozygous; ASD-autism spectrum disorder; FMF-familial Mediterranean fever; ED-ectodermal dysplasia; rows 1–3 strong evidence to support autism susceptibility; rows 4–6 lists published pathogenic variants with excellent clinical correlation. Based on review of WGS raw data, *MEFV* variants are in *cis*. Note: *VKORC1* and *CYP2C9* variants described in the text are polymorphisms and thus not represented in the rare variant table above.

## Data Availability

The data presented in this study are available in the supplemental materials of this manuscript submission.

## Supplementary Materials

The following are available online at https://www.mdpi.com/2075-4426/11/1/21/s1, Table S1: Detailed Sequence Variant Spreadsheet, Table S2: Detailed Structural Variant Spreadsheet.
